# The WRKY Family Transcription Factor GmWRKY72 Represses Glyceollin Phytoalexin Biosynthesis in Soybean

**DOI:** 10.3390/plants13213036

**Published:** 2024-10-30

**Authors:** Jie Lin, Ivan Monsalvo, Hyejung Kwon, Sarah Pullano, Nik Kovinich

**Affiliations:** Department of Biology, Faculty of Science, York University, 4700 Keele St., Toronto, ON M3J 1P3, Canada; lj0215@yorku.ca (J.L.); imm1802@my.yorku.ca (I.M.); hkwon96@my.yorku.ca (H.K.); pullans@my.yorku.ca (S.P.)

**Keywords:** *Glycine max*, transcription factor, transcriptional repressor, phytoalexin, glyceollin

## Abstract

Phytoalexins are plant defense metabolites that are biosynthesized transiently in response to pathogens. Despite that their biosynthesis is highly restricted in plant tissues, the transcription factors that negatively regulate phytoalexin biosynthesis remain largely unknown. Glyceollins are isoflavonoid-derived phytoalexins that have critical roles in protecting soybean crops from the oomycete pathogen *Phytophthora sojae*. To identify regulators of glyceollin biosynthesis, we used a transcriptomics approach to search for transcription factors that are co-expressed with glyceollin biosynthesis in soybean and stilbene synthase phytoalexin genes in grapevine. We identified and functionally characterized the WRKY family protein GmWRKY72, which is one of four WRKY72-type transcription factors of soybean. Overexpressing and RNA interference silencing of *GmWRKY72* in the soybean hairy root system decreased and increased expression of glyceollin biosynthetic genes and metabolites, respectively, in response to wall glucan elicitor from *P. sojae*. A translational fusion with green fluorescent protein demonstrated that GFP-GmWRKY72 localizes mainly to the nucleus of soybean cells. The GmWRKY72 protein directly interacts with several glyceollin biosynthetic gene promoters and the glyceollin transcription factor proteins GmNAC42-1 and GmMYB29A1 in yeast hybrid systems. The results show that GmWRKY72 is a negative regulator of glyceollin biosynthesis that may repress biosynthetic gene expression by interacting with transcription factor proteins and the DNA of glyceollin biosynthetic genes.

## 1. Introduction

Plant immunity consists of consecutive overlapping signaling and defense processes that are highly conserved in the plant kingdom. The immune response is initiated by pattern recognition receptors (PRRs) at the surface of the plant cell. PRRs recognize pathogen-associated molecular patterns (PAMPs) or plant cell damage-associated molecular patterns (DAMPs) and initiate pattern-triggered immunity (PTI). Upon recognition of PAMPs or DAMPs, PRRs transduce the immune signal by phosphorylating proteins, such as mitogen-activated protein kinases (MAPKs), and consecutive, partially overlapping signaling and defense processes ensue. These include protein phosphorylation cascades, the production of reactive oxygen species (ROS), calcium ion signaling, ubiquitin-mediated protein degradation, and hormone signaling, which ultimately converge upon the expression and activation of transcription factor proteins that regulate the transcription of genes involved in defense processes [1,2,3,4]. These processes include the production of metabolites such as cell wall lignin, callose, and phytoalexins.

**Figure 1:**
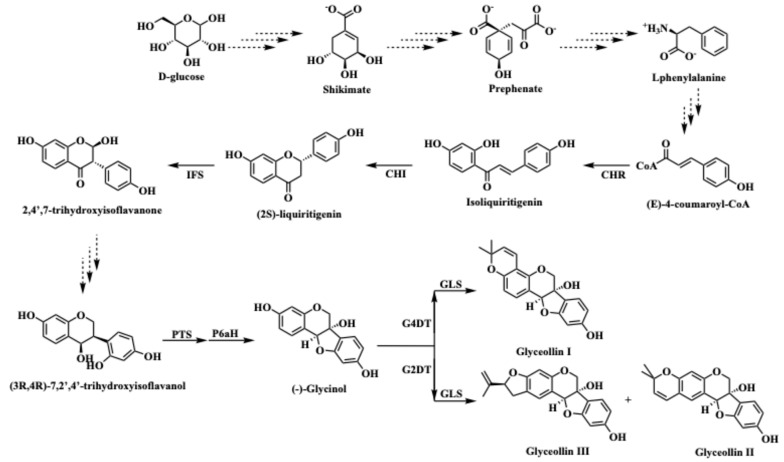
The glyceollin biosynthetic pathway in soybean. CHR, chalcone reductase; CHI, chalcone isomerase; IFS, isoflavone synthase; PTS, pterocarpan synthase; P6αH, dihydroxypterocarpan-6α-hydroxylase; G4DT, trihydroxypterocarpan dimethylallyltransferase; G2DT, dimethylallylpyrophosphate:trihydroxypterocarpan dimethylallyl transferase; GLS, glyceollin synthase.

Phytoalexins are specialized metabolites that are expressed in response to pathogens. Phytoalexins are an important component of plant immunity because they have critical roles in the resistance to many agriculturally important pathogens. For example, glyceollins have critical roles in protecting soybean (*Glycine max*) from the causal agent of Asian soybean rust, namely *Phakopsora pachyrhizi*, and the stem and root rot pathogen *Phytophthora sojae*. Purified glyceollins are toxic to *P. sojae* and *P. pachyrhizi* in vitro [5,6]. Some pathogens have evolved effectors that directly inhibit glyceollin biosynthesis [7]. Compelling evidence that glyceollins are essential for soybean’s immunity against *P. sojae* came from several genetic studies. Glyceollins are biosynthesized from the 5-deoxyisoflavonoid intermediate daidzein and their biosynthesis requires at least 21 enzymes, including chralcone reductase (CHR), isoflavone synthase (IFS), isoflavone reductase (IFR), pterocarpan synthase (PTS), and pterocarpan 6-α-hydroxylase (PTS) (Figure 1). RNAi silencing of *IFS* genes in the soybean hairy root system reduced the biosynthesis of glyceollins and several 5-deoxyisoflavonoids and resulted in susceptibility to *P. sojae* [8]. Overexpressing *IFR* in soybean seedlings increased glyceollin amounts and enhanced resistance to *P. sojae* [9]. Finally, by silencing the transcription factor *GmMYB29A2* in variety Williams 82, which is resistant to Race 1 *P. sojae*, and overexpressing the same gene in the susceptible variety Williams, we found that compatibility and incompatibility coincided with reduced and enhanced accumulations of glyceollin I specifically, and not the other glyceollins or 5-deoxyisoflavonoids [10].

To date, several transcription factors have been identified that regulate glyceollin biosynthesis in soybean. The NAC family transcription factor GmNAC42-1 directly activates the expression of several glyceollin biosynthetic genes in response to the PAMP from *P. sojae* named wall glucan elicitor (WGE) [11]. GmMYB29A2 directly binds the DNA of *GmNAC42-1* and several glyceollin biosynthetic genes. Overexpressing and RNAi silencing experiments in the soybean hairy root system demonstrated that *GmMYB29A2* positively regulates the expression of those genes in response to WGE and provides non-race-specific resistance to *P. sojae* [10]. Simultaneously overexpressing *GmNAC42-1* and *GmMYB29A2* in the soybean hairy root system fails to activate glyceollin biosynthesis in the absence of elicitation [12]. This is despite the fact that both proteins directly interact with the promoters of several glyceollin biosynthetic genes in the yeast one-hybrid (Y1H) and by electrophoretic mobility shift assay (EMSA) systems. Overexpressing *GmMYB29A2* activates almost all glyceollin biosynthetic genes; however, overexpressing *GmNAC42-1* results in almost no glyceollin gene upregulation in the absence of an elicitor treatment [12]. Thus, we hypothesized the existence of negative regulators that directly block the transactivation activity of GmNAC42-1 in the absence of elicitation.

The biosynthesis of phytoalexins is highly diverse, with different plant lineages biosynthesizing unique molecules via distinct metabolic pathways. Early studies aimed at identifying phytoalexin transcription factors identified unrelated proteins. Since these unrelated proteins were regulating distinct phytoalexin biosynthetic pathways in different plant lineages, this led to the notion that phytoalexin transcription factors are as diverse as the biosynthetic pathways that they regulate. However, we recently discovered a positive regulator of glyceollin biosynthesis in soybean, namely GmNAC42-1, which is the homolog of the *Arabidopsis* transcription factor ANAC042 (also known as JUNGBRUNNEN1, or JUB1) [11]. ANAC042 positively regulates the expression of the indole alkaloid phytoalexin camalexin in *Arabidopsis* [13]. We also found that the transcriptional activator of glyceollin biosynthesis, GmMYB29A2, and the negative regulator, GmMYB29A1, are soybean’s closest homologs to MYB15, which directly activates scopoletin phytoalexin and pathogen-inducible monolignol biosynthesis in *Arabidopsis* [10,14]. Our recent review of phytoalexin gene regulation in *Arabidopsis*, which included functional comparisons with transcription factors in other plant species, suggested that the transcription factors ANAC042, MYB15, MYB72, ERF1, and WRKY33, and the JAZ1 protein, may be part of a ‘core’ phytoalexin regulatory network that is conserved yet regulates distinct phytoalexin pathways in different plant lineages [15]. More research is needed to understand whether these transcription factors and others have conserved roles in regulating phytoalexin biosynthesis across plant species. Particularly, the negative regulation of phytoalexin biosynthesis remains poorly understood.

Glyceollins exhibit impressive anticancer and other pharmaceutical activities in mammalian models and human cell cultures [16,17,18]. They even inhibit the proliferation and growth of cells and tumors of triple-negative breast cancer (TNBC), a cancer type that is resistant to conventional chemotherapeutics and thus is in need of new therapies. Glyceollins, like other phytoalexins, are expressed only transiently and in low amounts by plants in response to pathogens. Due to the clinical importance of phytoalexins such as taxol [19,20,21] and prospective pharmaceuticals such as glyceollin I and their important roles in agriculture, there has been increasing interest in understanding how phytoalexin biosynthesis is regulated and can be enhanced in plants [19,21,22]. WRKY33 from *Arabidopsis* has a central role in regulating camalexin biosynthesis and is perhaps the most extensively characterized phytoalexin transcription factor. It was first identified as a protein that interacts with MAP kinase 4 (MPK4) substrate (MKS1), implicating it in the regulation of microbial defenses [23]. T-DNA loss-of-function mutants were later found to have increased susceptibility to the necrotrophic fungal pathogen *Botrytis cinerea* or the bacterial pathogen *Pseudomonas syringae [24]*. Then wrky33 mutants were found to be almost completely deficient in camalexin biosynthesis following infection with *B. cinerea* or *P. syringae* [25,26]. Molecular aspects of WRKY33’s post-transcriptional regulation and transcriptional targets were later characterized. The phosphorylation of different residues of WRKY33 by CPK5/CPK6 and MPK3/MPK6 increases the DNA binding ability of the WRKY33 protein and enhances its transactivation activity, respectively [27]. Phosphorylation of WRKY33 by MPK3/MPK6 is mediated by SUMOylation, and disruption of the SUMOylation process inactivates WRKY33 activity [28]. Finally, the physical interaction into transcriptional complexes with the transcription factor ETHYLENE RESPONSE FACTOR1 (ERF1) was found to synergistically activate the expression of camalexin biosynthetic genes by ethylene, jasmonate, and MAPK signaling pathways [29]. Other WRKY transcription factors that positively regulate phytoalexin biosynthetic genes include the following: GaWRKY1, an activator of gossypol biosynthesis in cotton [30], OsWRKY10, an activator of diterpenoid phytoalexins and resistance to *Magnaporthe oryzae* in rice [31], and ZmWRKY79, an activator of terpenoid phytoalexin biosynthesis in *Zea mays* and resistance to *Rhizoctonia solani* in *Nicotiana benthamiana* [32].

Soybean is an ancient polyploid that has undergone two genome duplications approximately 59 and 13 million years ago [33]. Of the 188 WRKY proteins, 17 have been functionally characterized from soybeans [34]. This contrasts the 53 out of 75 that have been characterized from the model plant *Arabidopsis*. Soybean WRKY genes have roles in positively regulating plant growth and development [35], seed size [36], and tolerance to drought, salt, and aluminum [37,38], while negatively regulating phosphorus tolerance [39]. In soybeans, several WRKYs regulate resistance to important pathogens. Soybean somatic embryos undergoing silencing of GmWRKY27 (Glyma15g00570), GmWRKY139 (Glyma13g44730), GmWRKY56 (Glyma08g23380), and GmWRKY106 (Glyma07g02630) showed a higher number of lesions than wildtype when infected with *Phakopsora pachyrhizi* [40]. The soybean hairy root system has been an efficient tool for establishing the function of genes [41]. Transgenic soybean hairy roots overexpressing GmWRKY136 (Glyma.14G103100), GmWRKY53 (Glyma.19G094100), or GmWRKY86 (Glyma.13G117600) displayed increased resistance to soybean cyst nematode (SCN) [42]. Further, silencing GmWRKY40 (Glyma.08G143400) in the soybean hairy root system caused enhanced susceptibility to *P. sojae* [43]. Treating soybean hairy roots with WGE has been used to characterize the genes involved in mediating the phytoalexin response to *P. sojae* since the ‘pulsed’ response stimulated by WGE is very similar to that observed upon *P. sojae* infection [10,44,45]. WRKY family transcription factors clearly have important roles in regulating pathogen defense in soybean and phytoalexin biosynthesis in other plant species, yet none have been identified that regulate glyceollin phytoalexin biosynthesis. Here, we conducted a transcriptomics approach to identify a WRKY gene candidate involved in regulating glyceollin biosynthesis in soybean. We then characterized its function using the well-established soybean hairy root gene transformation system [41] in addition to its gene and protein targets using yeast hybrid systems. Our results characterize GmWRKY72 as a negative regulator of glyceollin biosynthesis.

## 2. Results

### 2.1. RNA-Seq Identifies GmWRKY72 as a Candidate Regulator of Glyceollin Biosynthesis

**Figure 2:**
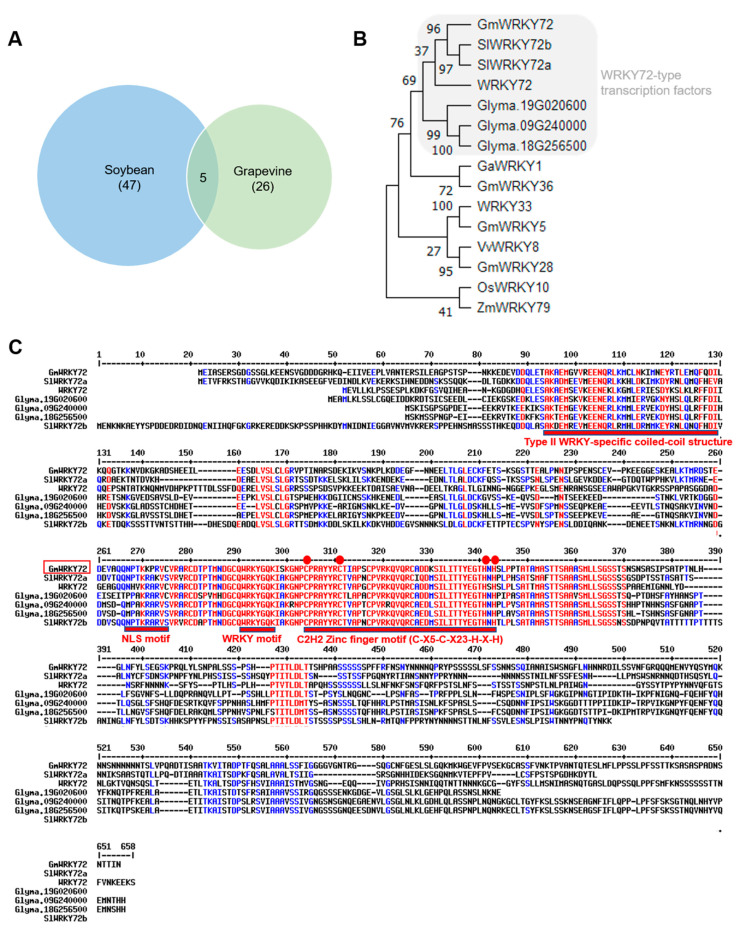
Identification and protein similarity analysis of GmWRKY72. (**A**) Number of transcription factors in soybean that were up-regulated by pH 3.0 medium or WGE and down-regulated by dehydration that were co-expressed with grapevine *VviSTS* genes (*p* < 0.05) by RNA-seq. (**B**) Cluster analysis of deduced amino sequences of GmWRKY72 and other closely related WRKY transcription factors. (**C**) Multiple alignments of GmWRKY72 and WRKY72 proteins from soybean, *Arabidopsis*, and tomato. *Glycine max*: GmWRKY72 (Glyma.17G097900), Glyma.09G240000, Glyma.18G256500, Glyma.19G020600. *Arabidopsis thaliana*: WRKY72 (At5G15130), *Solanum lycopersicum*: SlWRKY72a (GU017421), SlWRKY72b (GU017422), are from GeneBank. Identical and similar amino acids were in red and blue, respectively. Conserved motifs are underlined in red.

To identify candidate genes that regulate glyceollin biosynthesis, we searched for transcription factors that were consistently co-expressed with glyceollin biosynthetic genes in all four of our RNA-seq datasets. The datasets represented the transcriptome changes of Harosoy 63 seedlings exposed to acidity and dehydration stresses, which exhibit prolonged (week-long) inductive or suppressive effects on glyceollin biosynthesis, respectively [11], and Williams 82 hairy roots and Harosoy 63 seeds treated with wall glucan elicitor (WGE) from the oomycete pathogen *P. sojae* [10]. Forty-seven (47) transcription factor genes were consistently up- and down-regulated with glyceollin biosynthesis in all four datasets (Supplemental Table S1). Our recent studies have suggested that transcription factors that regulate glyceollin biosynthesis in soybeans are homologous to the regulators of stilbene biosynthesis in grapevine [15]. To further narrow down our list of candidates, we searched for homology among our 47 candidates and the 26 transcription factors that were co-expressed with stilbene phytoalexin genes in grapevine [46]. This identified five soybean transcription factor genes (Table 1) (Figure 2A). Two of the five had been characterized previously. *GmNAC42-1* and *GmHSF6-1* were found to positively regulate glyceollin biosynthesis in soybean hairy roots by RNAi and gene overexpression experiments and directly bind the promoter regions of several glyceollin biosynthetic genes in the yeast one-hybrid system [11,12]. *GmNAC42-2* is an uncharacterized paralog of *GmNAC42-1.* Their predicted proteins exhibit 85.8% amino acid similarity. GmMYB215 is uncharacterized and is predicted to encode 73.3% amino acid similarity compared to GmMYB29A2, which is a positive regulator of glyceollin biosynthesis and *Rps1* R-gene-mediated resistance to *P. sojae* [10].

The last candidate gene is predicted to encode a WRKY family protein that had no reported association with regulating phytoalexin biosynthesis. We cloned its coding sequence (CDS) from the cDNA of WGE-elicited Williams 82 hairy roots and confirmed that its nucleotide sequence is the same as Wm82.a2.v1 gene ID Glyma.17G097900. A BLAST search found that its predicted amino acid sequence is most similar to the WRKY72-type transcription factors of tomato (*Solanum lycopersicum*) and *Arabidopsis* (Figure 2B). These factors were required for R gene Mi-1-mediated resistance and basal defense against several pathogens in tomatoes and basal defense against several pathogens in *Arabidopsis* [47]. The predicted protein has the most amino acid similarity compared to SlWRKY72a (49.3%) (Supplemental Table S2). Thus, we named the corresponding gene *GmWRKY72*. A BLAST search of GmWRKY72 against the soybean proteome followed by a cluster analysis found that it is one of four soybean proteins that have greater similarity to WRKY72-type transcription factors than it does to the nearest soybean homolog, GmWRKY86 (Figure 2B). These WRKY72-type transcription factors form a cluster distinct from characterized phytoalexin regulators from cotton (i.e., GaWRKY1), *Arabidopsis* (WRKY33), rice (OsWRKY10), *Zea mays* (ZmWRKY79), and positive regulators of resistance to soybean cyst nematode (SCN), namely GmWRKY5/28/36/62/154 (Figure 2B).

*GmWRKY72* has an 1800-bp coding sequence (CDS) that spans 4543-bp of chromosome 17 of the soybean genome. The CDS is predicted to encode a protein of 600 amino acids that has a molecular mass of 64.9 kDa and an isoelectric point of 5.6. Sequence analysis revealed that the GmWRKY72 protein shared several conserved motifs with other WRKY72-type proteins: (1) It harbors an approximate 60-amino acid WRKY domain that contains the conserved amino acid sequence (WRKYGQK), (2) A coiled-coil structure at its N-terminus that is specific to type II WRKY transcription factors [48], and (3) A zinc-finger motif (C-X4–5-C-X22–23-H-X1-H) adjacent the WRKY domain that indicates that GmWRKY72 belongs to Group IIc of the WRKY family (Figure 2C) [49]. The predicted nuclear localization sequence (NLS) of GmWRKY72 harbored several amino acid differences compared to other WRKY72-type proteins. Given that the functions of WRKY72-type proteins remain poorly understood, we decided to investigate further the potential connection between GmWRKY72 and the regulation of glyceollin biosynthesis.

### 2.2. GmWRKY72 Negatively Regulates Glyceollin Biosynthesis

**Figure 3:**
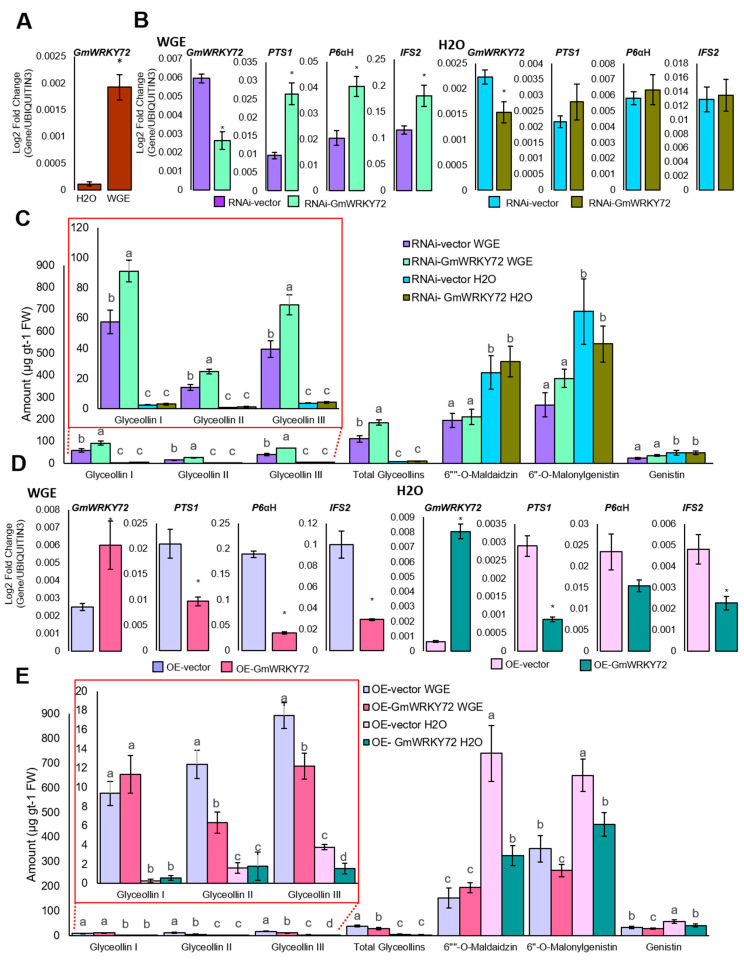
GmWRKY72 is a negative regulator of glyceollin biosynthesis in soybean. (**A**) Expression level of *GmWRKY72* gene in W82 hairy roots elicited for 24 h with H2O or WGE by qRT-PCR. The significance test was performed by paired Student’s *t*-test (*p* < 0.05), which is indicated by asterisks. Error bars indicate SE (n = 3). (**B**) Gene expression in hairy roots silencing *GmWRKY72* elicited for 24 h with WGE or mock (H_2_O) treatment. The significance test was performed by paired Student’s *t*-test (*p* < 0.05), which is indicated by asterisks. Error bars indicate SE (n = 3). (**C**) Amounts of glyceollins and isoflavonoid metabolites in soybean hairy roots undergoing RNAi silencing of *GmWRKY72* 24 h after treatment with WGE or H_2_O. The significance test was performed by single-factor ANOVA, Tukey post hoc test (*p* < 0.05, α = 0.05), which is indicated by different letters. Error bars represent SE (n = 5). (**D**) Transcript levels in hairy roots overexpressing *GmWRKY72* elicited for 24 h with WGE or mock (H_2_O) treatment. The significance test was performed by paired Student’s *t*-test (*p* < 0.05), which is indicated by asterisks. Error bars indicate SE (n = 3). (**E**) Amounts of glyceollins and isoflavonoids metabolites in hairy roots overexpressing *GmWRKY72* elicited for 24 h with WGE or mock (H_2_O) treatment. The significance test was performed by single-factor ANOVA, Tukey post hoc test (*p* < 0.05, α = 0.05), which is indicated by different letters. Error bars represent SE (n = 5).

WRKY72-type transcription factors have roles in positively regulating basal immunity in *Arabidopsis* and tomato, but their mechanism of action and the pathways that they regulate remain unknown. To determine whether GmWRKY72 exhibits pathogen-responsive expression, as indicated by our RNA-seq data, we conducted qRT-PCR on soybean hairy roots that were treated with WGE from the pathogen *P. sojae*. *GmWRKY72* was expressed at trace levels in the absence of an elicitor and was up-regulated 17.2-fold at 24 h after WGE treatment (Figure 3A), which has been shown to be the peak time of glyceollin biosynthesis in the hairy root system [10]. To investigate whether GmWRKY72 has a role in regulating glyceollin biosynthesis, we generated hairy roots harboring an RNA interference (RNAi) construct that encoded a hairpin dsRNA identical to a 298 bp region of exon 1 of *GmWRKY72*. We also generated roots that overexpress the *GmWRKY72* CDS via the constitutive cauliflower mosaic virus promoter. If GmWRKY72 regulates glyceollin biosynthesis, RNAi and gene overexpressor roots should have altered levels of glyceollin biosynthesis.

RNAi silencing *GmWRKY72* 2.3-fold in WGE-treated hairy roots resulted in the upregulation of glyceollin biosynthetic gene transcripts. Specifically, the transcript levels of *PTS1*, *P6αH*, and *IFS2* were up-regulated 2.7-, 2.0-, and 1.6-fold, respectively (Figure 3B). This was accompanied by a 1.7-fold increase in total glyceollin metabolite amounts (Figure 3C). By contrast, the metabolite levels remained unchanged for the phytoalexin genistein and the non-phytoalexin isoflavonoids 6″-*O*-malonyldaidzin and 6″-*O*-malonylgenistin (Figure 3B), suggesting that the silencing GmWRKY72 specifically affected glyceollin biosynthesis without impacting other isoflavonoids upon elicitation. In mock (H_2_O)-treated hairy roots, a 1.4-fold silencing of *GmWRKY72* did not affect the expression of glyceollin biosynthesis genes or metabolite levels (Figure 3B,C). Thus, the effects of silencing GmWRKY72 were specifically observed when the roots were elicited, as this is when GmWRKY72 expression is typically up-regulated. To test for off-target silencing, we measured the expressions of the three GmWRKY72 paralogs in the RNAi roots by qRT-PCR. Glyma.19G020600 and Glyma.09G240000 were up-regulated in both WGE- and mock-treated samples. By contrast, the expression of Glyma.18G256500 did not change under mock treatment and was only 0.7-fold reduced under WGE (Supplementary Figure S1). Thus, the effects observed in RNAi roots are likely specific to silencing GmWRKY72. To gain further insight into the function of GmWRKY72, we then overexpressed its CDS in the hairy root system.

Overexpressing *GmWRKY72* 2.4-fold in WGE-treated hairy roots down-regulated the expression of glyceollin biosynthetic genes. Specifically, the expression of *PTS1*, *P6αH*, and *IFS2* was reduced by 2.2-, 5.5-, and 3.4-fold, respectively (Figure 3D). It also reduced the total glyceollin metabolite levels by 1.4-fold (Figure 3E). Ectopically overexpressing *GmWRKY72* 12.2-fold in mock-treated hairy roots down-regulated the expression of glyceollin biosynthesis gene transcripts *PTS1*, *P6αH*, and *IFS2* 3.3-, 1.5-, and 2.1-fold, respectively (Figure 3D). Glyceollin metabolite levels are minimal in the absence of elicitation and were not altered in mock-treated hairy roots. However, the ectopic overexpression reduced the levels of 6″-*O*-malonyldaidzin and 6″-*O*-malonylgenistin by 2.3- and 1.4-fold, respectively (Figure 3E). This may be explained by IFS enzyme being required for the biosynthesis of all isoflavonoids and glyceollins.

GmWRKY72 is expressed at trace levels in the absence of an elicitor and is highly up-regulated by WGE. Since silencing and overexpressing GmWRKY72 results in increased and decreased glyceollin biosynthesis, respectively, specifically during WGE treatment, this suggests that GmWRKY72 functions as a negative regulator of the glyceollin biosynthesis upon elicitation. Whether it does so directly by binding biosynthetic genes or indirectly by physically interacting with glyceollin transcription factors or another mechanism remained important mechanisms to investigate.

### 2.3. GmWRKY72 Localizes to the Nucleus and Directly Binds Glyceollin Transcription Factors and Biosynthesis Gene Promoters

**Figure 4:**
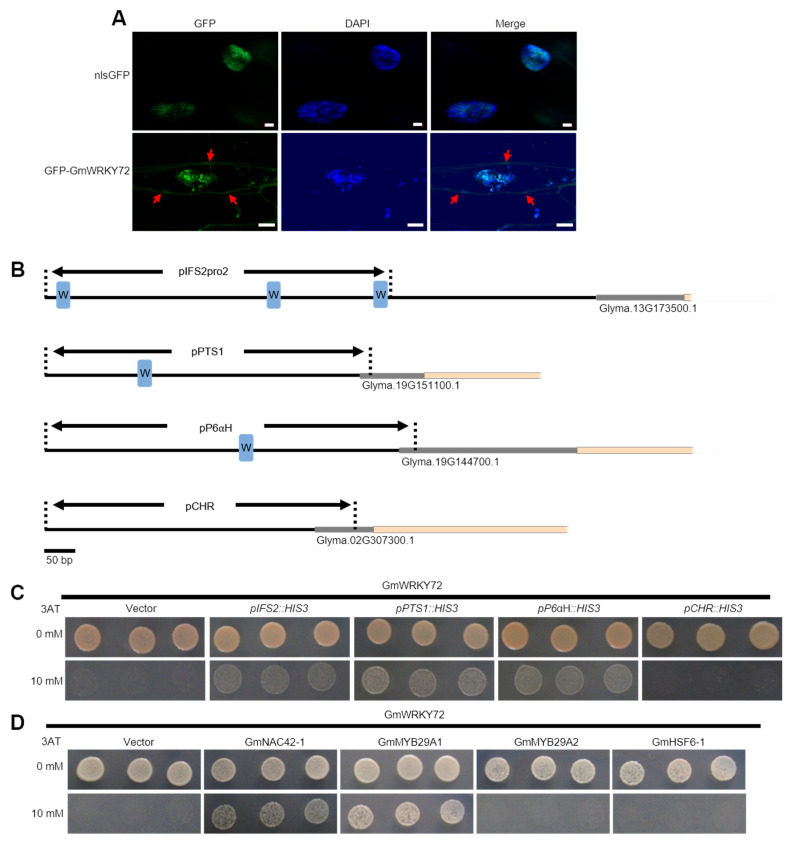
Characterization of the subcellular localization, protein–protein interactions (PPIs), and protein–DNA interactions (PDIs) of the GmWRKY72 protein. (**A**) Fluorescence microscopy of GmWRKY72 translationally fused to green fluorescent protein (GFP) in transgenic W82 hairy roots. Green fluorescent protein harboring an N-terminal nuclear localization sequence (NLS-GFP) was used as positive control, and DAPI (6 µg/mL) images indicate nuclear staining. Red arrows indicate some localization of GFP-GmWRKY72 to the cell periphery. Bars are 2 µm and 5 µm, respectively. (**B**) Schematic diagram demonstrating promoter fragments of *IFS2*, *PTS1*, *P6αH*, and *CHR* used for yeast one-hybrid assays. W-box elements with sequence 5′-TTGAC-3′ (blue boxes). (**C**) Yeast one-hybrid analysis of strain YM4271 transformed with GmWRKY72-Gal4AD and *pIFS2::HIS3*, *pPTS1::HIS3, pP6*αH*::HIS3*, *pCHR::HIS3* and on SD-Leu-His (SD-L-H). Growth on SD-L-H + 0 mM 3-aminotriazole (3AT, top rows) are plating controls, and SD-L-H + 20 mM 3AT (bottom rows) indicate positive PDIs. (**D**) Yeast two-hybrid analysis testing for PPIs between GmWRKY72 and GmNAC42-1, GmMYB29A1, GmMYB29A2, and GmHSF6-1. Yeast strain PJ69-4a was transformed and plated on SD-L-T and SD-L-T-H ± 3AT, respectively. Growth on SD-L-T-H+ 0 mM 3AT (top rows) are plating control; growth on SD-L-T+H + 40 mM 3AT (bottom rows) indicates positive PPIs. pDEST-GADT7 was used as the empty ‘Vector’ control.

To determine whether the subcellular localization of the GmWRKY72 protein is consistent with its putative role as a transcription factor, we cloned its CDS downstream of an N-terminal GFP tag and expressed the translational fusion in soybean hairy roots using the constitutively active CaMV-35S promoter. nGFP-GmWRKY72 localized mainly to punctate spots in the nucleus, as shown by co-localization with DAPI fluorescence (Figure 4A). It also demonstrated relatively lower levels of localization to the periphery of the cell (red arrows, Figure 4A). By contrast, the control translational fusion of GFP with an N-terminal nuclear localization sequence co-localized exclusively with DAPI in the nucleus. Thus, GmWRKY72 is predominantly localized to the nucleus, suggesting a potential role as a transcription factor.

To test whether the GmWRKY72 protein directly binds the promoters of glyceollin biosynthesis genes, the CDS was also cloned downstream of the GAL4 activation domain and expressed in yeast harboring ~500 bp segments of the *IFS2, PTS1,* and *P6αH* promoters. Each promoter segment was predicted to encode at least one W-box element with the sequence 5′-TTGAC-3′ [50] (Figure 4B). For comparison, we also included in our analysis a segment of the *CHR* promoter that lacked the W-box. GmWRKY72 interacted with the *IFS2, PTS1*, and *P6αH* promoters that had one or more predicted W-box elements and failed to interact with the *CHR* promoter that lacked the element (Figure 4C). This suggests that GmWRKY72 directly binds the regulator regions of some glyceollin biosynthetic genes.

Our recent studies have identified several transcription factors that either positively or negatively regulate glyceollin biosynthesis [10,11,12]. Since we have identified several transcription factors that directly bind and regulate the expression of the same glyceollin biosynthetic genes [10,11,12], this raises the question of whether these proteins physically interact. To investigate whether the GmWRKY72 protein physically interacts with any of the characterized glyceollin transcription factors, we conducted yeast two-hybrid analyses. We introduced GmWRKY72 harboring an N-terminal translational fusion of the yeast Gal4 activation domain (Gal4AD) into yeast strains that express glyceollin transcription factors as N-terminal translational fusions of the Gal4 DNA-binding domain (Gal4BD). Gal4AD-GmWRKY72 physically interacted with Gal4BD-GmNAC42-1 and Gal4BD-GmMYB29A1, but not with Gal4BD-GmMYB29A2 or Gal4BD-GmHSF6-1 (Figure 4D). Thus, the GmWRKY72 protein physically interacts with glyceollin transcription factors, suggesting the combinatorial regulation of biosynthetic genes by transcriptional complexes.

## 3. Discussion

### 3.1. GmWRKY72 Is a Negative Regulator of Glyceollin Biosynthesis in Soybean

WRKY transcription factors play important roles in the growth and development of plants. They are also central regulators of the transcriptional responses to environmental stresses and microbial pathogens [51,52]. The molecular mechanisms remain unclear for most WRKYs involved in defense against pathogens. However, an increasing number of WRKYs have been identified that directly regulate the transcription of phytoalexin biosynthetic genes [53]. Phytoalexins have critical roles in defending plants in agriculture. For example, glyceollins have an essential role in mediating incompatibility between the soybean genotypes that carry the *R* gene *Rps1k* and Race 1 *P. sojae* [10,54]. Further, overexpressing GmMYB29A2, a positive regulator of glyceollin biosynthesis, rendered the susceptible variety Williams incompatible with Race 1 *P. sojae.* To date, WRKY transcription factors have been found to regulate the biosynthesis of several phytoalexins in various plant species, yet none have been identified to regulate phytoalexin biosynthesis in legumes.

To identify candidate transcriptional regulators of glyceollin biosynthesis, we compared four RNA-seq datasets of soybeans undergoing differential regulation of glyceollin biosynthetic genes and two transcriptome compendia of genes that are co-expressed with stilbene synthase homologs in grapevine. This identified five candidate glyceollin transcription factors (Table 1, Figure 2A). The WRKY family gene *GmWRKY72* was confirmed by qRT-PCR to be highly up-regulated at the time of maximum glyceollin biosynthesis in response to WGE from *P. sojae* (Figure 3A). We isolated its cDNA from WGE-elicited hairy roots and found a translational fusion with GFP localized to the nucleus of soybean hairy root cells (Figure 4A). Overexpression and RNA interference (RNAi) silencing of *GmWRKY72* decreased and increased the expression of glyceollin biosynthetic genes and metabolites, respectively, in response to WGE (Figure 3). The promoters of the differentially regulated biosynthetic genes harbored W-box elements. The GmWRKY72 protein bound those promoters in the yeast one-hybrid system but did not bind the *CHR* promoter that lacked the W-box element (Figure 4B,C). The GmWRKY72 protein physically interacted with GmNAC42-1, a positive regulator of glyceollin biosynthesis, and GmMYB29A1, which is a negative regulator, in the yeast two-hybrid system (Figure 4D). Collectively, the results suggest that GmWRKY72 acts as a repressor of glyceollin biosynthesis that likely functions by interacting with the regulatory regions of glyceollin biosynthetic gene DNA and transcription factor proteins.

The question arises as to why a repressor of glyceollin biosynthesis would be co-expressed with glyceollin biosynthetic genes. Insight into this came from our RNAi experiments, which demonstrated that silencing GmWRKY72 results in higher levels of glyceollin transcripts and metabolites upon elicitation with WGE (Figure 3). By contrast, RNAi silencing did not change the expression levels of glyceollins in mock-treated samples. These results suggest that the function of GmWRKY72 is to limit glyceollin biosynthesis during elicitation rather than keeping it off in the absence of an elicitor treatment. So why would plants evolve genes to limit phytoalexin biosynthesis? One possibility is that excessively high concentrations of phytoalexins may be toxic to the host plant cell [55], and avoidance of toxicity may be preferred in tissues such as soybean roots that typically do not exhibit the hypersensitive response (i.e., apoptosis) when synthesizing phytoalexins [56,57]. Further, phytoalexin production in planta is transient, which requires biosynthesis to be activated by positive regulators and presumably turned off by negative ones. The typical plant immune response to microbial pathogens is characterized by a series of consecutive processes, such as the production of reactive oxygen species (ROS), defense proteins, cell wall lignins, phytoalexins, etc., which happen in succession with some overlap. Thus, it is tempting to speculate that the magnitude and duration of each process would have to be tightly controlled by regulators such as GmWRKY72 to ensure an adequate distribution of cellular resources for all processes that are required to achieve a robust immune response.

To our knowledge, there are only two other examples of WRKYs that negatively regulate phytoalexin biosynthesis other than GmWRKY72. BnWRKY15 physically interacts with the W-box of the promoter of *BnWRKY33* to repress its transcription and camalexin biosynthesis in vivo [58]. BnWRKY15 overexpression also increased the susceptibility of *B. napus* to *S. sclerotiorum*. VvWRKY8 from grapevine physically interacts with the transcriptional activator VvMYB14 and blocks its interaction with stilbene synthase gene promoters to negatively regulate the biosynthesis of resveratrol [52]. There are several examples of WRKYs in various plant species that negatively regulate plant resistance to pathogens. The *Arabidopsis* loss-of-function mutant and gene overexpressor lines of WRKY25 exhibit increased and reduced resistance to *P. syringae* strain ES4326 [59,60]. WRKY48 negatively regulates plant defense against *P. syringae* [61]. WRKY40 reduces the expression of early defense-induced genes to negatively regulate PTI [62]. HvWRKY1/2 suppresses basal resistance to virulent *Blumeria graminis* [63] and powdery mildew [64]. For both GmWRKY72 and VvWRKY8, it remains to be determined whether their function in planta negatively or positively contributes to the resistance against microbial pathogens.

### 3.2. Subfunctionalization Among WRKY72-Type Transcription Factors

Suppressing the expression of *SlWRKY72a* and *SlWRKY72b* by virus-induced gene silencing resulted in a clear reduction of the R gene Mi-1-mediated resistance and basal defense against potato aphid, root-knot nematode (RKN), and *Pseudomonas syringae* in tomato [47]. By contrast, T-DNA insertion mutants demonstrated that WRKY72 is not required for resistance against *P. syringae* in *Arabidopsis*, but is required for full basal defense against RKN and the oomycete pathogen *Hyaloperonospora arabidopsidis*. The molecular processes regulated by tomato and *Arabidopsis* WRKY72s remain unknown. Microarray experiments from the same study found that 3050 *Arabidopsis* genes are up-regulated by *H. arabidopsidis*, and 363 of those genes are not up-regulated in the WRKY72 T-DNA mutant [65]. Our analysis of those gene lists found that all phytoalexin marker genes investigated were up-regulated by *H. arabidopsidis*, including *WRKY33*, *PAD3*, *CYP71A12*, *CYP71B15*, *CYP79B2*, *MYB15,* or *4CL*. However, those marker genes were not up-regulated (or down-regulated) in the WRKY72 T-DNA mutant. While it is noteworthy that the T-DNA dataset had some limitations, for example, the WRKY72 gene itself was not found to be differentially expressed compared to the wildtype, the results do raise the possibility that WRKY72 in *Arabidopsis* may not regulate phytoalexin biosynthesis. Since WRKY72s are required for resistance to *P. syringae* in tomato but not *Arabidopsis* [47,65], the results suggest that WRKY72 genes have undergone some degree of subfunctionalization. Soybean is an ancient polyploid (palaeopolyploid) that has undergone several genome duplications, with nearly 75% of its genes occurring in multiple copies [33]. Our BLAST search and protein clustering identified three soybean paralogs of GmWRKY72 (Figure 2). We have previously observed that paralogs (i.e., GmMYB29A2 and GmMYB29A1) have roles in positively and negatively regulating glyceollin biosynthesis [10]. It should be investigated whether other WRKY72-type transcription factors have roles in positively or negatively regulating phytoalexin biosynthesis in plants and soybean resistance to *P. sojae*.

### 3.3. Growing Evidence of Phytoalexin Transcription Factor Complexes

Phytoalexins have critical roles in mediating plant resistance against agriculturally important pathogens. Some phytoalexins are also important clinical pharmaceuticals or prospective medicines that show potent, unconventional activities in pre-clinical research. Yet, phytoalexins are produced only transiently and in low amounts in plants. Thus, understanding the regulation of phytoalexin biosynthesis is of great importance to enhancing their accessibility for agriculture and medicine. Phytoalexin production in plants is mainly regulated at the level of transcription of the biosynthetic genes by transcription factor proteins. Earlier studies identified distinct transcription factors that regulate different phytoalexin biosynthetic pathways [66]. However, recent studies have found that some of the previously identified transcription factors, for example, NAC42, MYB15, and WRKY33, regulate distinct phytoalexin biosynthetic pathways in different plant lineages [10,11,51]. There is growing evidence that different phytoalexin transcription factors regulate the same biosynthetic gene promoters and that some physically interact to form transcriptional complexes. Moreover, negative regulators have been identified that may disrupt the formation of transcriptional complexes and/or redirect complexes to bind other gene promoters that are not involved in phytoalexin biosynthesis.

VvWRKY8 from grapevine physically interacts with the transcriptional activator VvMYB14 and blocks its interaction with stilbene synthase gene promoters to negatively regulate the biosynthesis of resveratrol [52]. OsWRKY45 interacts with OsWRKY62 to positively regulate the diterpenoid phytoalexin factor (OsDPF), thereby activating diterpenoid phytoalexin biosynthesis in rice [67]. ERF1 interacts with and depends on WRKY33 to upregulate camalexin biosynthetic genes. The interaction is proposed to be a point of convergence between the ethylene/JA and MPK3/MPK6 signaling pathways to synergistically induce camalexin biosynthesis in *Arabidopsis* [29]. *Vitis quinquangularis* factors VqNAC44 and VqMYB15 physically interact in the yeast two-hybrid and bimolecular fluorescence complementation systems [68]. While VqNAC44 did not directly bind stilbene synthase (*STS*) gene promoters, it enhanced *STS* gene expression and stilbene metabolite levels when co-infiltrated with VqMYB15 into grape leaves, suggesting that the interaction is required for VqNAC44 to regulate stilbene biosynthesis.

Here, by yeast one-hybrid and genetic engineering experiments using the soybean hairy root system, we found that GmWRKY72 binds and downregulates the transcription glyceollin biosynthetic genes, including *IFS2* (Figure 3 and Figure 4). We have previously found that the glyceollin transcriptional factors GmNAC42-1, GmMYB29A2, and GmHSF6-1 all bind and regulate the *IFS2* gene promoter [10,11,12]. Here, we found that GmWRKY72 physically interacts with GmNAC42-1 in the yeast two-hybrid system. Since GmWRKY72 physically interacts with the GmNAC42-1 and the IFS2 gene promoter, it is tempting to speculate that this provides further evidence that phytoalexin biosynthetic genes are regulated by complexes of transcription factors. Our future research will focus on testing the phytoalexin transcriptional complex hypothesis in planta.

## 4. Materials and Methods

### 4.1. Plant Materials and Growth Conditions

Seeds of Williams 82 (W82) soybean (*Glycine max*) used in this study were collected from the greenhouse at York University (September 2023). The dried seeds were surface sterilized with 70% isopropanol and 10% bleach, then rinsed three times with sterilized MilliQ H_2_O as described by Lin et al. [41]. After that, soybean seeds were placed on the germination co-culture (GC) medium, followed by dark culture (25 °C) for 3 days and dim light culture (22 °C) for 4 days before transformation.

### 4.2. Cloning and Plasmid Constructs

The full-length CDS of GmWRKY72 were PCR amplified from the complementary DNA of W82 hairy root (HR) treated with wall glucan elicitor (WGE, 24 h) using specific primers (Table S3). The PCR amplicons were inserted into entry vector pDONR221 using BP clonase (Invitrogen, Burlington, ON, Canada). For generating GmWRKY72 transgenic soybean HRs, entry clone (GmWRKY72-pDONR221) was LR (Invitrogen, ON, Canada) recombined into the destination vector pMDC7. For yeast one-hybrid and yeast two-hybrid assays, GmWRKY72-pDONR221 was LR subcloned into pDEST-GADT7 (pAD) and pBD-GAL4-GW-C1 (pBD). To assess subcellular localization, GmWRKY72 entry clone was LR recombined into pGWB6, which has a GFP tag at the N-terminus of the insert. For RNAi silencing, a 298-bp region corresponding to GmWRKY72 CDS was amplified from cDNA, BP recombined into pDONR221 and LR recombined into the RNAi destination vector pANDA35HK. IFS2, PTS1, and P6αH promoter regions were inserted into pGG vector via BsaI site and LR subcloned into the destination vector pMW#2.

### 4.3. Transcriptome Data Analysis

For soybean RNAseq data, the analysis of four biological duplicates of acidity (pH 3.0) and dehydration-treated W82 seedlings, WGE-treated W82 seeds, and hairy roots were reported in [10,11]. Both datasets are available in the Gene Expression Omnibus (GEO) database at the National Center for Biotechnology Information (NCBI) under the accession numbers GSE131686 and GSE221901. For the grapevine data, the analysis of two separate transcriptome compendia (microarray data sets and RNA-seq data sets) were reported in [46].

For analysis of the transcription factor (TF) co-expressions, TFs were considered putative glyceollin regulators when they met the requirement that was up-regulated by pH 3.0 or WGE and down-regulated by dehydration. Venn diagram was used to show the emergence of selected soybean TF homologs in grapevine that were co-expressed with stilbene phytoalexin biosynthetic genes.

### 4.4. Hairy Root Transformation and Elicitation

*Agrobacterium rhizogenes* strain NCPPB2659 (K599) containing the empty vectors, overexpression, or RNAi constructs, was used to transform sterilized soybean cotyledons using a previously described protocol [41]. Briefly, K599 harboring the plasmid of interest was grown on Luria–Bertani (LB) medium (Bioshop, Burlington, ON, Canada) plates containing hygromycin (Gold Biothechnology, Olivettee, MO, USA) and kanamycin (50 mg/L; overnight) (Gold Biothechnology, Olivettee, MO, USA) for overexpressing and spectinomycin (100 mg/L; overnight) (Gold Biotechnology, Olivettee, MO, USA) for silencing. Agrobacteria cells were resuspended in ½ PB buffer and adjusted to an OD600 of 0.5–0.8 before use. Seven-day-old soybean cotyledons were infected by three 5-mM-deep cuts with *A. rhizogenes* suspension. The inoculated cotyledons were cut side down and co-cultured under dim light for 3 days using GC medium (100 μM Acetosyringone, Cayman Chemical, Ann Arbor, MI, USA). After that, cotyledons were transferred into hairy root growth (HRG, Timentin = 500 mg/L, Caisson Lab, Logan, UT, USA) medium. Secondary roots that are more than 3 cm were transferred to antibiotic plates (kanamycin and/or hygromycin, 50 mg/L) and grown for 5 days. Positive HRs were harvested and cut into ~1 cm fragments for elicitation. Each biological replicate was paired in order to be subjected to metabolic extraction and flash-frozen for RNA isolation.

### 4.5. Isoflavonoids Analyses

Metabolite isolation was performed according to Farrell et al. [69]. Briefly, tissues were smashed in an MM400 mixer mill (Retsch, Newtown, CT, USA) at 30/s frequency, added 80% ethanol (1 µL mg^−1^ fresh tissue), and smashed again. Centrifuge at maximum speed for 3 min, then incubated in the freezer (−20 °C) overnight. The following day, centrifuge again and filter the supernatant through 0.2 µm in a microcentrifuge tube. Samples were analyzed using UPLC-PAD (Thermo Scientific, Waltham, MA, USA) and identification and quantification of each isoflavone component using Chromeleon 7.2.10 software (Thermo Scientific, Waltham, MA, USA).

### 4.6. RNA Extraction and Gene Expression Measurements

Total RNA was isolated from soybean HRs using HiPure Total RNA Mini Kit (GeneBio System, Burlington, ON, Canada) following the manufacturer’s protocol. Complementary DNA was obtained using DNA synthesis kit (GeneBio System, Burlington, ON, Canada) following the manufacturer’s protocol. qRT-PCR was conducted on a Bio-Rad CFX96 machine (Bio-Rad Laboratories, Mississauga, ON, Canada) using GB-AmpTM Sybr Green qPCR mix (GeneBio System, Burlington, ON, Canada). The thermal cycling was as follows: initial denaturation at 95 °C for 3 min, followed by 40 cycles of 95 °C for 5 s and 60 °C for 30 s, and a melt curve analysis was included from 65 °C to 95 °C. *GmUbi3* served as the internal reference for the transcripts in soybean. The comparative CT method: expression = 2^−[Ct(gene) − Ct(UBIQUITIN3)]^ was used to analyze qPCR data. Primers used in this study are listed in Supplemental Table S3.

### 4.7. Subcellular Localization

Soybean HRs transformed into nlsGFP-GmWRKY72-pGWB6 or nlsGFP-pGWB6 (negative control) were harvested while the secondary roots were approximately 1 cm. Samples were mounted on the 10% glycerol and stained by 4′,6-diamidino-2-phenylindole (DAPI, 6 μg/mL) (Cayman Chemical, Ann Arbor, MI, USA). Five roots per genotype were analyzed. Confocal laser microscopy (LMS 700, Carl Zeiss, Oberkochen, Germany) was used to observe the GFP fluorescence and Zen Black v2.3 SP1 software was used to modify the image. Excitation and emission spectra were 488 nm and 500–550 nm for GFP and 405 nm and 358–461 nm for DAPI, respectively.

### 4.8. Yeast Hybrid Assays

For yeast one-hybrid, the recombinant plasmid pAD-GmWRKY72 and promoters (pMW#2-IFS2, pMW#2-PTS1, and pMW#2-P6αH) were transformed into the YM4271 yeast (*Saccharomyces cerevisiae*), respectively. SD-Leu-His medium was used to select the positive transformants. SD-Leu-His plates containing 3-amino-1,2,4-triazole (3AT; Fisher Scientific, Waltham, MA, USA) were used for screening protein–DNA interactions (PDIs).

For yeast two-hybrid, the prey pAD-GmWRKY72 and the baits (pBD-GmNAC42-1, pBD-GmMYB29A1, pBD-GmMYB29A2, pBD-GmHSF6-1) were transformed into the PJ69-4a yeast strain, respectively. Transformants were selected on SD-Leu-Trp plates. Positive protein–protein interactions (PPIs) were determined by co-transformed PJ69-4a yeast growth on SD-His-Leu-Trp medium that contained 3AT.

The pAD empty vector was used as negative control for both yeast hybrid assays.

### 4.9. Statistical Analysis

Statistics were conducted using the Tukey post hoc test and one-way single-factor ANOVA to assess for any significant statistical differences between groups. Paired comparisons of the qPCR data analysis were analyzed by Student’s *t*-test using Excel software v2016. It was considered significant with a *p*-value less than 0.05.

### 4.10. Accession Numbers

GmWRKY72, Glyma.17G097900; GmNAC42-1, Glyma.02G284300; GmMYB29A1, Glyma.10G006600; GmHSF6-1, Glyma.03G135800; PTS1, Glyma.19G151100; P6αH, Glyma.19G144700; IFS2, Glyma.13G173500; CHR, Glyma.02G307300.

## Figures and Tables

**Table 1:** Homologous soybean and grapevine transcription factors that are co-expressed with their respective phytoalexin biosynthetic genes.

Soybean Gene Name	Grapevine Gene Name	TF Family	Soybean Gene ID	Grapevine Gene ID	Reference
*GmNAC42-1*	*VviNAC36*	NAC/S6	Glyma.02G284300	VIT_12s0028g00860	[11]
*GmNAC42-2*	*VviNAC36*	NAC/S6	Glyma.14G030700	VIT_12s0028g00860	-
*GmHSF6-1*	*VviHsfB3a*	HSF/A	Glyma.03G135800	VIT_08s0007g08750	[12]
*GmMYB215*	*VviMYB15*	R2R3-MYB/S2	Glyma.10G180800	VIT_05s0049g01020	-
*GmWRKY72*	*VviWRKY53*	WRKY/llb	Glyma.17G097900	VIT_17s0000g05810	-

## Data Availability

All data supporting the findings of this study are available within the paper and Supplementary Materials.

## Supplementary Materials

The following supporting information can be downloaded at: https://www.mdpi.com/article/10.3390/plants13213036/s1: Figure S1: off-target gene expression in hairy roots silencing *GmWRKY72* 24 h after treatment with WGE or H_2_O; Table S1: list of selected transcription factors up-regulated by pH 3.0 medium or WGE and down-regulated by dehydration; Table S2: percent amino acid similarity to GmWRKY72; Table S3: list of oligonucleotides used in this study.
