# Supplementary material for: The WRKY Family Transcription Factor GmWRKY72 Represses Glyceollin Phytoalexin Biosynthesis in Soybean
Source: Plants (Basel). 2024 Oct 30;13(21):3036. doi: 10.3390/plants13213036 (PMC11548433; doi:10.3390/plants13213036)
Supplement: Supplementary file 1 [file plants-13-03036-s001.zip › Figure S1 Off-target gene expression levels.pptx]

## Slide 1
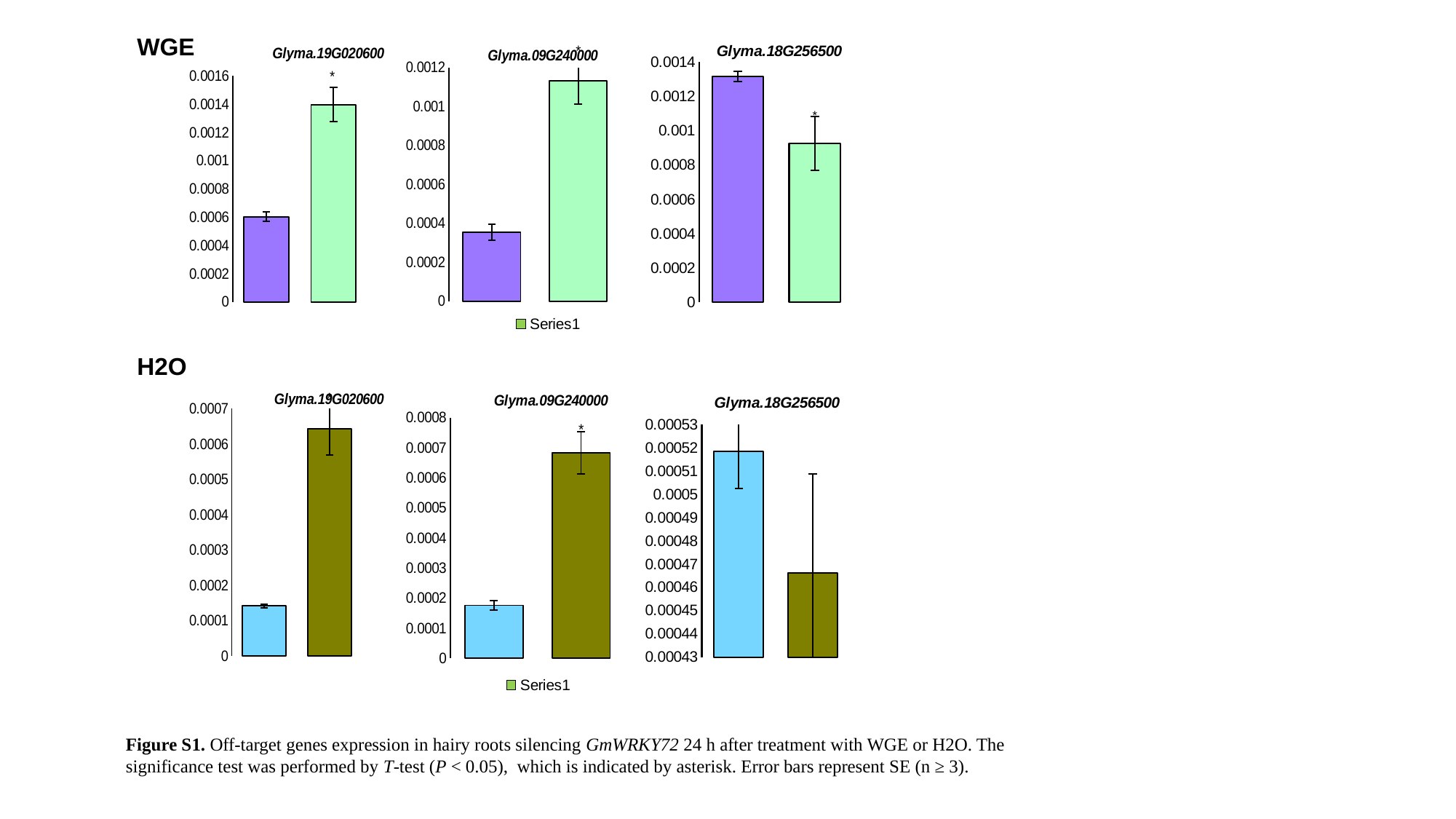

WGE
### Chart: Glyma.18G256500
| Category | |
|---|---|
| RNAi-vector | 0.0013171511636551614 |
| RNAi-GmWRKY72 | 0.0009266869990406028 |
### Chart: Glyma.09G240000
| Category | |
|---|---|
| RNAi-vector | 0.00035418136131563243 |
| RNAi-GmWRKY72 | 0.001132492613208202 |
### Chart: Glyma.19G020600
| Category | |
|---|---|
| RNAi-vector | 0.0006037025128830338 |
| RNAi-GmWRKY72 | 0.0013980474303971018 |H2O
### Chart: Glyma.18G256500
| Category | |
|---|---|
| RNAi-vector | 0.0005185876995363504 |
| RNAi-GmWRKY72 | 0.000466098977533332 |
### Chart: Glyma.19G020600
| Category | |
|---|---|
| RNAi-vector | 0.0001420306188928982 |
| RNAi-GmWRKY72 | 0.0006435948314208817 |
### Chart: Glyma.09G240000
| Category | |
|---|---|
| RNAi-vector | 0.00017575287944473586 |
| RNAi-GmWRKY72 | 0.0006827413104585143 |Figure S1. Off-target genes expression in hairy roots silencing GmWRKY72 24 h after treatment with WGE or H2O. The significance test was performed by T-test (P < 0.05), which is indicated by asterisk. Error bars represent SE (n ≥ 3).
